# Preoperative lipiodol marking and its role on survival and complication rates of CT-guided cryoablation for small renal masses

**DOI:** 10.1186/s12894-017-0199-1

**Published:** 2017-01-18

**Authors:** Fumiya Hongo, Yasuhiro Yamada, Takashi Ueda, Terukazu Nakmura, Yoshio Naya, Kazumi Kamoi, Koji Okihara, Yusuke Ichijo, Tsuneharu Miki, Kei Yamada, Osamu Ukimura

**Affiliations:** 1 Department of Urology, Kyoto Prefectural University of Medicine, 465 Kajii-cho, Kamigyo-ku, Kyoto, 602-8566 Japan; 2 Department of Radiology, Kyoto Prefectural University of Medicine, Kyoto, Japan

**Keywords:** Ablation, Cryoablation, Lipiodol marking, Renal cell cancer, Small renal mass

## Abstract

**Background:**

Partial nephrectomy for small renal masses (SRM) may be useful for preserving renal function, but is technically more difficult than radical nephrectomy. Cryoablation may be performed under local anesthesia. The objective of the present study is to assess the safety and therapeutic efficacy of cryoablation with lipiodol marking for SRM.

**Methods:**

Cryoablation therapy was performed on 42 patients under local anesthesia. Their median age was 74 years (31–91). The median tumor diameter was 21 mm (10–42). Responses to the treatment were evaluated using modified Response Evaluation Criteria in Solid Tumors (mRECIST) by contrast-enhanced CT. In six patients (14.3%) for whom it was not possible to use contrast medium, plain CT findings were assessed according to Response Evaluation Criteria in Solid Tumors (RECIST).

**Results:**

Twenty-nine (69%) and five (12%) patients achieved complete responses (CR) and partial responses (PR), respectively, while four (10%) and four (10%) patients each had stable disease (SD) and progressive disease (PD) after the first course of therapy. A second course of cryoablation therapy with lipiodol marking was performed on three out of four patients with PD after the first course of therapy, and resulted in a total of 32 patients achieving CR (76%). Four (36.4%) out of 11 patients for whom lipiodol marking was not conducted had PD, whereas none of the 31 patients for whom lipiodol marking was conducted had PD. All grade complications were reported in 11 (24.4%) patients while grade 3 in two (4.4%) patients. 11 (24.4%) A significant difference was observed in postoperative hemorrhagic events in all grades (18% in patients undergoing cryoablation without lipiodol marking vs. 0% in patients undergoing cryoablation without lipiodol marking).

**Conclusions:**

Although further studies involving more patients are needed in order to evaluate long-term results, cryoablation therapy appears to be a useful treatment option for SRM. Preoperative marking with lipiodol was helpful for improving complication and survival rates with cryoablation.

## Background

Renal function-preserving surgery has recently been recommended as a treatment for small renal cancer [1–3]. Percutaneous cryoablation therapy, which includes thermal ablation, for humans was initially reported by Uchida [4]. Laparoscopic cryoablation was subsequently conducted, and favorable outcomes were reported [5]. In Japan, cryoablation has been covered by national health insurance since 2011. We started to perform percutaneous cryoablation therapy for SRM in March 2013, and herein report our initial experience with this procedure.

Computed tomography (CT)- or magnetic resonance imaging (MRI)-guided puncture is conducted in cryoablation therapy. One of the advantages of CT-guided puncture is that it provides a broader space for puncture than MRI-guided puncture; however, it is more difficult to recognize tumor margins with CT-guided puncture than with MRI-guided puncture. Plain CT-guided puncture is performed in our hospital. In some patients with submerged tumors or tumor margins that are difficult to recognize, marking with lipiodol is conducted prior to cryoablation therapy.

Lipiodol is a lipid-soluble contrast material that is used for lymphangiography [6], hysterosalpingography [7], and transcatheter arterial chemoembolization (TACE) of hepatocellular carcinoma [8]. Since lipiodol remains in place for several days, it is easy to localize nodules using X-ray or CT fluoroscopy during surgery. In the present study, we examined the efficacy of cryoablation therapy for SRM and the usefulness of preoperative lipiodol marking.

## Methods

### Patients

In March 2013, our hospital started to perform cryoablation therapy on patients who were not indicated for radical surgery under general anesthesia because of active double cancer or complications or on those who did not wish to undergo surgery due to the presence of only one kidney or for some other reason. A preoperative staging imaging evaluation (chest to abdominal CT) was routinely performed on all patients. We retrospectively examined the efficacy of this procedure, adverse events, and post-treatment changes in renal function. Pre- and postoperative serum creatinine levels and adverse events in patients aged 75 years or older were compared with those in patients aged 74 years or younger. The present study was conducted in accordance with the Principles of Helsinki. This study protocol was approved by Institutional Review Board of Kyoto Prefectural University of Medicinw. The Ethics board approval number was ERB-C-54-1. All patients included in this study provided informed consent for cryosurgery, accompanying standard care and the use of their data in research.

### Cryoablation methods

The treatment plan was made by performing CT before ablation. A CryoHit® device (Galil Medical USA; Hitachi Medical Corporation, Japan) was employed. IceSeed® or IceRod® needles were used in accordance with the tumor diameter. One to three needles were used for ablation as one IceSeed® for less than 10 mm, 2 IceSeeds® for 10–12 mm, 3 IceSeeds® or 2 IceRods® for 13–20 mm, 3 IceRods® for 21–30 mm, and 4 IceRods® for 31–40 mm. The cryoprobe was introduced under CT fluoroscopic guidance (Vigor Laudator, Toshiba Medical System, Tokyo, Japan) after local anesthesia had been administered by a subcutaneous injection of 1% lidocaine.

The tumor site was cooled with argon gas and thawed with helium gas. The cryoablation area was monitored at appropriate times during puncture or cryoablation. Two cycles of cryoablation were then performed, with the first cycle typically lasting 10–15 min and the second 10 min. Passive thaw was performed between the ablation cycles, and active thaw was performed after the second cycle.

When the tumor was adjacent to peripheral organs, such as the intestinal tract, hydrodissection with physiological saline was performed in order to avoid injury. Transdiaphragmatic puncture with an artificial pneumothorax was conducted for transthoracic puncture. When the tumor was adjacent to the renal pelvis, a ureteral catheter was inserted in some patients, and the tumor site was perfused with warm physiological saline to avoid injury to the renal pelvic mucosa.

As a rule, percutaneous tumor biopsy using 18-gauge Max-Core® (BARD, USA) was performed for a histopathological diagnosis before or at the time of cryoablation because the tumor histology and grade of preoperative biopsy predicted the oncological outcomes of renal cryoablation [9]. Local anesthesia and the prophylactic administration of antibiotics were permitted as combined and supportive therapies.

Transarterial lipiodol marking was performed 1–3 days before cryoablation therapy when difficulties were associated with identifying the tumor location on plain CT. Transfemoral visceral arteriography was conducted using a standard angiographic approach. Selective catheterization of the tumor-feeding arteries was performed under fluoroscopic guidance. After confirming the presence of the catheter tip in the branches of the renal arteries feeding the tumor, lipiodol (Laboratoire Guerbet, Roissy, France) was manually injected (range, 0.2–0.4 mL) under fluoroscopy to make a lipiodol spot.

### Evaluation of efficacy

Responses to the treatment were evaluated by performing contrast-enhanced CT after 6 months. Efficacy was evaluated based on the tumor response rate, namely, a complete response (CR) or partial response (PR), using the modified Response Evaluation Criteria in Solid Tumors (mRECIST) criteria [10, 11]. The mRECIST criteria incorporate amendments to the original RECIST criteria. Tumor responses were defined as: (i) CR: the disappearance of any intratumoral arterial enhancement in all target lesions; (ii) PR: at least a 30% decrease in the sum of diameters of viable (enhancement in the arterial phase) target lesions, taking the baseline sum of the diameters of target lesions as the reference; (iii) stable disease (SD): any cases that do not qualify for either PR or progressive disease (PD); (iv) PD: An increase of at least 20% in the sum of the diameters of viable (enhancing) target lesions, taking the smallest sum of the diameters of viable (enhancing) target lesions recorded since the treatment started as the reference. Efficacy was evaluated based on the tumor response rate, namely, CR or PR, using the mRECIST criteria.

### Complications

The Clavien Classification of Surgical Complications was used for surgically related complications [12].

### Statistical analysis

Relationships between clinicopathological characteristics and response rates were examined using the *χ*2 test. Changes in serum creatinine levels were examined using the *t*-test. Test results were considered significant at *P* < 0.05. All analyses were performed using JMP 10.0.2 (SAS®).

## Results

### Patients

Cryoablation therapy was performed on a total of 42 patients before December 2014 (Table 1). Their median age was 74 years (range, 31–91). The median tumor diameter was 24.1 mm (range, 10–42 mm).

**Table 1:** The characteristics and outcomes of patients underwent cryopablation with or without preoperativeliiodol marking

Preoperative lipiodol marking		(+) (*n* = 31)	(−) (*n* = 11)	*p* value
Mean age (year, range)	74 (31–91)	71.5 (31–86)	71.1 (49–91)	NS
Gender (%)				NS
Male	33 (79%)	27	6	
Female	9 (21%)	5	4	
Laterality (%)				NS
Right	21 (50%)	15	6	
Left	21 (50%)	16	5	
Tumor size (mm)	24.1 (10–42)	27.8 (10–42)	21.3 (15–34)	*p* < 0.05
Biopsy performed in 36/42 (85.7%)		28	8	NS
RCC	30 (87%)	24	6	
AML	1 (4%)	0	1	
Inappropriate sample	5 (9%)	4	1	
PD (%)	4 (36.4%)	0	4 (36.4%)	*p* < 0.001
Post ablative hemorrhagic event (%)	2 (18%)	0	2 (18%)	*p* < 0.05

Percutaneous renal biopsy was performed on 86% of patients (36/42), but was not mandatory. Biopsy data are shown in Table 1. A pathological diagnosis of renal cell cancer (RCC) was reached in 30 out of the 36 patients and benign tumor (AML) in one patient who underwent biopsy. In the other five patients, biopsy specimens were insufficient to make a pathological diagnosis.

### Response

Treatment responses were evaluated using mRECIST based on contrast-enhanced CT findings (Table 2). In six patients (14.3%) for whom it was not possible to use contrast medium, plain CT findings were assessed according to RECIST. After the first course of therapy, 29 (69%) and five (12%) patients achieved complete responses (CR) and partial responses (PR), respectively, while four (10%) and four (10%) patients each had stable disease (SD) and progressive disease (PD). A second course of cryoablation therapy with lipiodol marking was performed on three out of the four patients with PD after the first course of therapy. CR was achieved in two patients and PR in 1. Final treatment responses were evaluated in 42 patients, including three who underwent two courses of cryoablation therapy. CR and PR were achieved in 32 and five patients, respectively. SD and PD were noted in four and one patients, respectively. one patient proved to have AML. The technical success rate was 98%.

**Table 2:** Efficacy of cryoablation. In three of four patients with PD after the first therapy, second cryoablation therapy with lipiodol marking was performed

	No. of cases	CR	PR	SD	PD
mRECIST	36	32 (89%)	3 (8%)	0 (0%)	1 (3%)
RECIST	6	0 (0%)	2 (33%)	4 (67%)	0 (0%)
Total	42	32 (76%)	5 (12%)	4 (10%)	1 (2%)

### Complications

There were 11 episodes (24.4%) of complications during a total of 45 courses of cryoablation therapy regardless of the grade. Grade 3 or higher adverse events were observed in two patients (4.4%). Intra- and postoperative complications included fever, hematoma, hematuria, pleural effusion, hydronephrosis, and ureter perforation in 5, 1, 2, 1, 1, and 1 patient, respectively (Table 3). Grade 3 or higher adverse events were observed in two patients: G3a hydronephrosis and G3a ureteral injury. There were no lipiodol marking-related adverse events.

**Table 3:** Postoperative complication in 45 sessions of cryoablation according to Clavien-Dindo classification

	<75 yo (*n* = 21, 23 sessions)		≥75 yo (*n* = 21, 22 sesseions)		Total	
	All grade	Grade 3≤	All grade	Grade 3≤	All grade	Grade 3≤
	5 (22%)	1 (4%)	6 (27%)	1 (5%)	11 (24%)	2 (4%)
Fever	2 (9%)		3 (14%)		5 (11%)	
Hematoma	1 (4%)		1 (4%)		2 (4%)	
Hematuria	1 (4%)		1 (5%)		1 (2%)	
Pleural effusion			1 (5%)		1 (2%)	
Hydronephrosis	1 (4%)	1 (4%)			1 (2%)	1 (2%)
Ureter perforation			1 (5%)	1 (5%)	1 (2%)	1 (2%)

### Renal function after cryoablation

Postoperative renal function was investigated based on serum creatinine levels 3 months after the treatment. Preoperative serum creatinine levels were 0.95 ± 0.4 in patients aged 74 years or younger and 1.19 ± 0.61 in those aged 75 years or older. These values 3 months after surgery were 0.96 ± 0.46 and 1.20 ± 0.55, respectively. The rates of changes were −1 ± 10 and 3 ± 15%, respectively, which were not significantly different (*p* = 0.3282).

We conducted preoperative lipiodol marking before cryoablation therapy on 31 patients with guidance difficulties under plain CT. Baseline patient demographics and operative outcomes are listed in Table 1. No significance differences were observed in mean age (71.5 vs. 71.1), gender (male/female) (27/5 vs. 6/4), or tumor laterality (15/16 vs. 6/5). On the other hand, significant differences were detected in tumor sizes (27.8 mm (10–42) vs. 21.3 (15–34) mm).

**Fig. 1:**
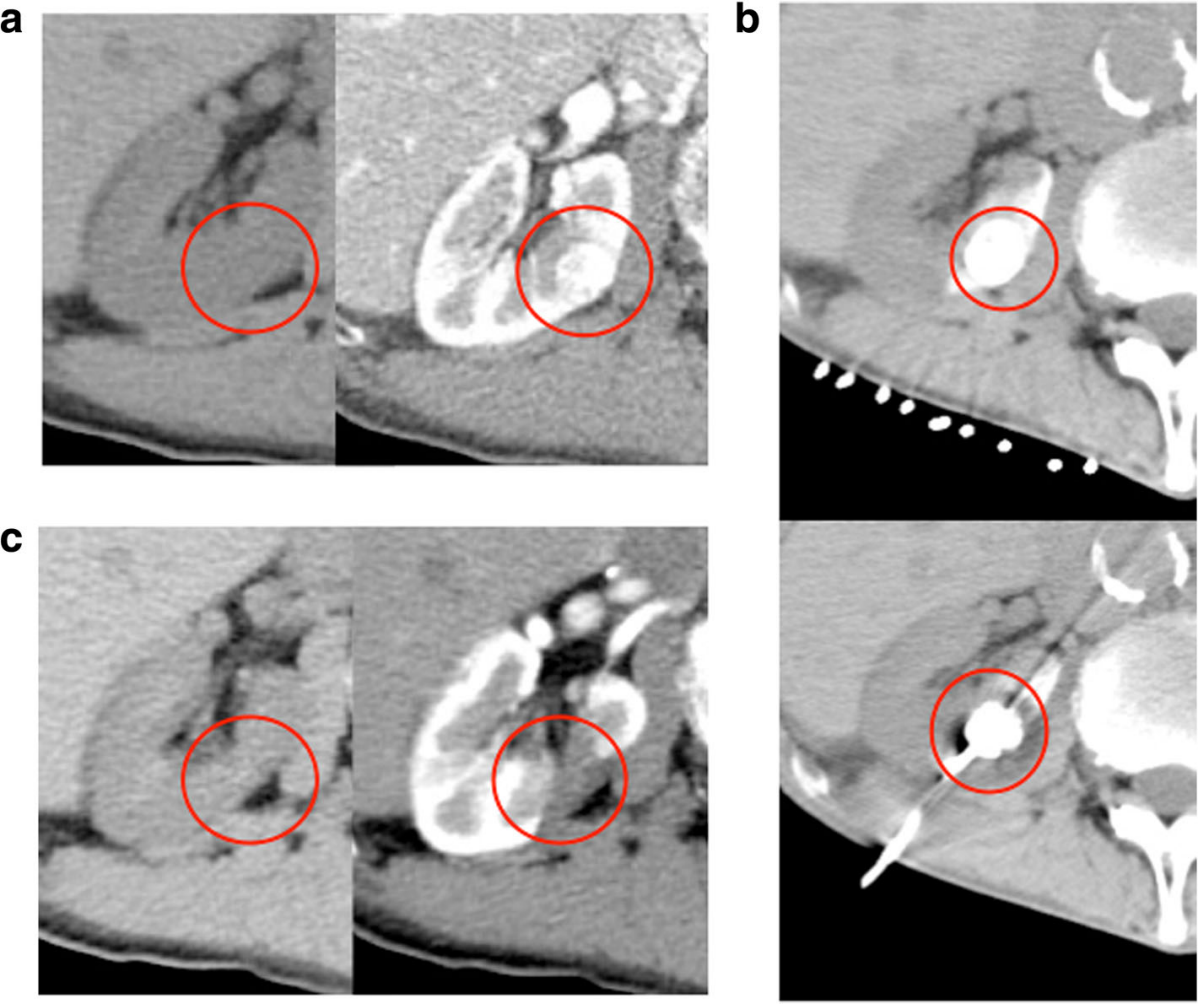
A case of cryoablation with preoperative lipiodol marking. The red circle indicates the primary tumor. **a** The right renal tumor was detected by a preoperative dynamic computed tomography (CT) scan. **b** The tumor was easily detected by intraoperative plain CT after lipiodol marking. **c** A postoperative CT scan showed no enhancement in the cryoablated area. A complete response was achieved by cryoablation according to the mRECIST criteria

A case of cryoablation therapy with preoperative lipiodol marking was shown (Fig. 1). The red circle indicates the primary tumor. (A) The right renal tumor was detected by a preoperative dynamic CT scan. (B) The tumor was easily detected by intraoperative plain CT after lipiodol marking. (C) A postoperative CT scan showed no enhancement in the cryoablated area. CR was achieved by cryoablation according to mRECIST.

Among all 42 patients, relapse was detected in four (36.4%) out of 11 patients for whom lipiodol marking was not conducted and was not observed in any (0%) of the 31 patients for whom lipiodol marking was conducted showed relapse, with a significant difference between with and without marking (*p* = 0.01). Moreover, a significant difference was detected in postoperative hemorrhagic events (18% vs. 0%) (*p* < 0.05) (Table 1).

No deaths occurred within 1 month of cryoablation therapy. Although one patient died during the follow-up period, her death was not related to cryoablation therapy; she died of primary disease (malignant lymphoma) 20 months after cryoablation therapy.

Patient survival was evaluated at a mean follow-up time of 17 (range, 6–26) months (SD, 6.13 months). One- and 2-year overall survival rates were 100 and 94.4%, respectively.

## Discussion

Nephrectomy has been performed as a standard treatment for renal cancer for a long time. However, the detection rate of SRM has increased with recent advances in diagnostic imaging procedures. A paradigm shift in treatment approaches to renal masses is underway, leading the AUA to release guidelines for the management of clinical stage 1 renal masses in 2009 for the first time [1]. Partial nephrectomy or ablative therapy for T1a renal cancer may be useful for preventing nephrectomy-related chronic kidney disease (CKD) [13, 14]. In elderly patients or patients with comorbidities, who are likely have a lower estimated glomerular filtration rate (eGFR), rapid reductions in renal function have been implicated in early death [15]. Therefore, not only partial nephrectomy, but also ablative therapy including cryoablation therapy, which may be performed under local anesthesia without renal ischemia, thereby facilitating the preservation of renal function, may be useful for patients with comorbidities and the elderly. In the present study, the impact of cryoablation therapy on renal function in patients aged 75 years or older was not significant.

Treatment options for SRM include ablation therapy. In our hospital, RFA therapy has been performed as advanced medical care and its usefulness has been reported [16, 17]. However, in Japan, RFA for renal tumors is not yet covered by national health insurance. Therefore, cryoablation therapy, which is covered by national health insurance, is primarily performed in our hospital.

CT- or MRI-guided puncture is optional as a percutaneous approach. Plain semi-real-time CT-guided puncture is performed in our hospital. However, the major limitation of plain CT is the difficulty associated with the localization of the tumor, when the tumor resembles the renal parenchyma, and, importantly, patients indicated for ablative therapy may have renal dysfunctions that are a contraindication for the frequent intraoperative use of a CT contrast agent. In order to overcome these limitations, we evaluated the usefulness of lipiodol marking to identify the tumor center prior to percutaneous cryoablation with plain CT guidance. In the selected patients with submerged tumors or those in whom the tumor margin was difficult to recognize, marking with lipiodol was conducted prior to cryoablation therapy. We previously reported the usefulness of preoperative lipiodol marking prior to the ablation of lung cancer [18]. The use of cryoablation therapy for renal cancer has facilitated accurate evaluations of target tumors, thereby improving the accuracy of the treatment. In contrast, cryoablation therapy may also be performed with the confirmation of tumor contours by administering contrast medium. However, the use of contrast medium needs to be avoided in patients with renal hypofunction. Furthermore, a previous study indicated that embolization prior to cryoablation therapy was useful for reducing the incidence of complications related to cryoablation therapy [19]. Regarding lipiodol marking, lipiodol with a gelatin sponge is transarterially infused. It may be useful for identifying the tumor location or its margin on CT-guided probe insertion and preventing hemorrhage-associated complications because it reduces intra-tumor blood flow.

When the tumor was adjacent to peripheral organs, such as the intestinal tract, hydrodissection [20], which is useful for avoiding thermal ablation-related intestinal injury, was performed. There were no intestinal injuries in any patients (0%).

In 115 tumors were treated using PCA, technical success rate was achieved in 97% by post-procedure CT (with and without IV contrast or MRI on POD 1). There was no evidence of local recurrence in 80 tumors that were followed for a mean of 13.3 months by CT imaging [21]. Kapoor et al. reported that the maximal and minimal percentages of cancer-specific survival were 100% and 84.3% in follow-ups of 11.4 months (median) and 64 months (mean), respectively, by reviewing a total of 2104 analyzed tumors from 2038 patients in the literature [22].

On the other hand, the timing of follow-ups after cryoablation therapy has not yet been established. One exception was the series by Gill and colleagues; the authors routinely performed biopsies 6 months post-CA. In this series, two out of 56 tumors were positive 6 months post-CA, at a rate of 3.6% [23]. In our hospital, follow-ups are performed using dynamic CT 1, 3, 6, 9, and 12 months after the treatment. In the present study, treatment responses were evaluated after 6 months. The success rate for the treatment was 98%, which was consistent with previous findings. When biopsies revealed no malignancy, follow-up CT was performed 6 and 12 months after the treatment.

Primary complications include hemorrhage. Hemorrhage or significant bleeding requiring blood transfusion has a reported incidence of 1–8% [24].

In the present study, postoperative hemorrhage was observed in two patients (4%) in whom it was impossible to discontinue anticoagulant therapy. However, these patients did not require blood transfusions. There were no cases of hemorrhage requiring blood transfusion (0%). This percentage is lower than that previously reported and may be attributed to the artery-embolizing effects of preoperative marking with lipiodol, which is conducted in our hospital where necessary, thereby decreasing the incidence of postoperative hemorrhage. Transcatheter renal arterial embolization with a mixture of ethanol and lipiodol for unresectable RCC has been reported [24], and this study is the first to demonstrate the efficacy of cryoablation with lipiodol marking. A previous study showed that embolization before cryoablation therapy reduced the rate of complications [25]. In the present study, there were no intestinal injuries. However, based on previous findings, we prepared preventive strategies, such as cryoablation therapy after hydrodissection, for patients for whom the anterior surface of the kidney was suspected to be in contact with the intestinal tract [26].

Regarding complications in the urinary tract, ureteral perforation was noted in one patient. However, the insertion of a Double J stent catheter for 6 months led to improvements. The thermal ablation of renal tumors in close proximity to or abutting the renal pelvis, ureteropelvic junction (UPJ), or proximal ureter represents a higher risk scenario for ureteral or collecting system injury, with resultant obstruction or renal loss [16]. Cryoablation is more likely to be recommended than RF ablation for the treatment of a renal tumor in proximity to the ureter because the former procedure has been shown to result in fewer urinary tract injuries than the latter, as demonstrated in a porcine model [27].

According to a recent review on treatments for localized renal masses in the United States, nephrectomy still accounts for a high proportion, but its rate has decreased. Partial nephrectomy and thermal ablation, such as cryoablation and RFA, have been indicated for an increasing number of patients [28]. This may also be the case in Japan in the future. Partial nephrectomy is now primarily performed as a renal function-preserving treatment; however, cryoablation therapy may be useful for elderly patients and those with complications. A percutaneous thermal ablation procedure for renal cancer appears to be a useful treatment option for SRM. In the present study, preoperative marking with lipiodol was helpful for achieving successful cryoablation.

There were several limitations in the present study. The total number of cases was too small to reach definite conclusions. In addition, complete preoperative histological information was unavailable, limiting the oncological outcomes. Furthermore, this was a retrospective study. Therefore, controlled randomized trials need to be designed that compare preoperative lipiodol marking followed by cryoablation and cryoablation without lipiodol marking.

## Conclusions

The results of the present study suggest that cryoablation with preoperative lipiodol marking improves the safety and success rate of CT-guided cryoablation for SRM. This approach was even shown to be useful for patients with renal dysfunctions, who were likely contraindicated for the intraoperative use of a contrast agent to visualize renal tumors.

## Abbreviations

CA: Cryoablation
CKD: Chronic kidney disease
CT: Computed tomography
eGFR: Estimated glomerular filtration rate
HCC: Hepatocellular carcinoma
Lipiodol: Ethiodized oil
mRECIST: Modified Response Evaluation Criteria in Solid Tumors
MRI: Magnetic resonance imaging
PCA: Percutaneous cryoablation
POD 1: Post operative day 1
RCC: Renal cell cancer
SRM: Small renal masses
TACE: Transcatheter arterial chemoembolization
UPJ: Ureteropelvic junction
